# Developing a practical tool for measuring parental vaccine hesitancy: A people-centered validation approach in Dutch

**DOI:** 10.1080/21645515.2025.2466303

**Published:** 2025-02-17

**Authors:** Daphne Bussink-Voorend, Jeannine L. A. Hautvast, Tim Wiersma, Reinier Akkermans, Marlies E. J. L. Hulscher

**Affiliations:** a Radboud Institute for Health Sciences, Primary and Community Care, Radboud University Medical Center, Nijmegen, The Netherlands; b IQ Health Science Department, Radboud University Medical Center, Nijmegen, The Netherlands

**Keywords:** Vaccine hesitancy, routine childhood vaccination, mixed-methods, validation, measurement, decision-making

## Abstract

Vaccine hesitancy (VH) about routine childhood vaccinations drives falling vaccine uptake rates. This is a major public health challenge and therefore important to monitor uniformly. The challenge is that different methods are used to measure VH and that, in non-English language, there may not be an unequivocal translation of VH terminology. We aimed to develop and validate a method to assess VH in a simple and self-reported manner, with a mixed-methods study using a people-centered approach with a diverse group of parents and illustrated in Dutch. In the quantitative part, parents were asked to rate experienced vaccine hesitancy on a 10-point Likert scale, using five differently worded Dutch translations for VH. We analyzed internal consistency with Cronbach’s alpha and correlation with the short parental attitudes about childhood vaccination (PACV-5) scale. A total of 532 parents participated in the survey. We found that the five worded alternatives measured the same construct, indicated by a Cronbach’s alpha of 0.95. The wording resembling ‘doubt’ showed highest correlation with the PACV-5 score (coefficient −0.525, *p* < .001). In the qualitative part of this study, we conducted cognitive interviews with 12 lower educated parents to evaluate the comprehensibility and interpretation of the question and VH translations. The findings reinforced and added to the quantitative findings that ‘doubt’ is the preferred wording. Based on the integrated results, we developed the Vaccine Hesitancy Assessment (VHA) tool. We encourage its use for monitoring purposes at the time of decision-making as this may provide a window of opportunity for decision-support interventions.

## Plain Language Summary

Children less often receive vaccinations due to vaccine hesitancy. This is a major health problem that we want to monitor. But, there are different ways to measure hesitancy and to translate hesitancy into other languages. This makes monitoring and comparison between countries difficult. In this study, we developed a simple measure for hesitancy in a self-reported manner. Input from parents played an important role. We used two methods to collect information: numeric information from questionnaires and views and preferences of parents from interviews. In this study, we looked at five possible translations for hesitancy in Dutch. In the questionnaires, parents were asked to score their level of hesitancy from 1 to 10 for each of the five translations. We then compared the scores for each different translation with each other and with another often used measure of vaccine hesitancy. A total of 532 parents completed the questionnaire. We found very similar scores for the hesitancy questions with different translations. But, the translation resembling ‘doubt’ in Dutch corresponded most with the often used measure for vaccine hesitancy. Additionally, we interviewed 12 parents about how they understood the sets of question and answer to score their level of hesitancy. We evaluated how easy this was to read and which translation matched best with their understanding. These findings were in line with the results from the questionnaire and gave directions for improvement. This resulted in a measure called the Vaccine Hesitancy Assessment (VHA) tool. This tool can be used to monitor vaccine hesitancy.

## Introduction

Increasingly, attention is being drawn to declining confidence in vaccination and declining vaccination rates in children.^1^ The non-acceptance of vaccination can endanger the health of a community through disease outbreaks, as illustrated by an increase in measles outbreaks across Europe and America.^2–4^ This threat is endorsed by the WHO, which included vaccine hesitancy (VH) in its list of top-10 threats to global health in 2019.^5^

During the COVID-19 pandemic, the topic of vaccination came under scrutiny and widespread misinformation about vaccination fueled hesitancy.^6^ Beliefs about the COVID-19 vaccine have also influenced parental perceptions about childhood vaccination.^7^ Compared to the pre-pandemic situation, parents still mostly have a positive intention to vaccinate their children.^8^ But, the choice to vaccinate seems less evident, as parents deliberate more when deciding about vaccination.^9^ In the context of these developments, it is important to understand the process that precedes the (non)acceptance of vaccination.

The Precaution Adoption Process Model (PAPM) takes a process-oriented view on how decisions are made that lead to behavior. This model consists of a series of successive stages that precede the adoption of preventive behaviors, such as vaccination. These stages describe the process of becoming aware, becoming engaged and coming to a decision. Once a decision is made, intended behavior may or may not be carried out, leading to the (non)acceptance of vaccination.^10–12^ How people pass through these stages varies from progressing quickly to not progressing to the next stage or regressing.^12^ In our prior work, we defined VH as a state of indecisiveness regarding a vaccine decision.^13^ This is best understood as having difficulty getting past the decision-making stage.

Recognizing these different stages helps to differentiate between people who have not given the topic of vaccination much thought or have not yet made a decision, versus people who already have formed their opinion and made a decision about vaccination. People in these different stages may respond differently to communication and may require a different approach.^12^ For example, parents with fixed views on vaccination are shown to be less receptive to vaccine messaging compared to parents with susceptible views.^14^ From this perspective, it is important to identify the group of parents with VH, who are in the process of decision-making and are struggling to get beyond that point.

Many efforts have already been undertaken to design instruments to measure VH at the individual level.^15^ In most cases, VH is assessed in a brief but highly heterogeneous manner with one to three questions.^13^ Other scales include, for example, the Parental Attitudes about Childhood Vaccination (PACV),^16^ the SAGE instrument,^16^ the Vaccine Hesitancy Scale,^17,18^ or the 5C scale.^19^ The comprehensiveness of these scales makes them less suitable to be practically applied to monitor VH. Additionally, the majority of these scales operationalize VH in a rather broad manner, also asking about attitudes and previous vaccination behavior.^13, 20^ For example, the 5C scale focuses on the psychological antecedents of vaccination, such as confidence, complacency, constraints, calculation, and collective responsibility.^19^ So far, none of the developed methods assessed VH exclusively as ‘being indecisive in a decision to vaccinate.’ Measuring VH accordingly would align with PAPM stage ‘undecided,’ and identifies a group with views about vaccination that may still be flexible and receptive to information and interaction.

Translating VH instruments into various non-English languages is another challenge to adequately measure VH and compare results across different geographical areas. Prior research shows that there may not be a commonly used and accepted term for VH in different European languages.^21^ In the case of VH, words matter and merely translating may not be enough. Involving the people whose hesitancy is being assessed is crucial to ensure that the instrument measures what it claims to measure, meets the views and preferences of its intended users and is suitable in everyday practice.^22^

In this paper, we describe the development and validation of a simple and concise assessment of VH, a state of indecisiveness regarding a decision about routine childhood vaccination, the Vaccine Hesitancy Assessment (VHA) tool. We apply a mixed-method to not only assess psychometric properties but also to understand and validate the findings through integration with qualitative data. Furthermore, we use a people-centered approach by including views and preferences of parents in the development process to ensure applicability in the local context. This process is illustrated in the Dutch context and language. As coverage rates for routine childhood vaccinations are declining in the Netherlands, a focus on the assessment of VH is needed, making the development of the VHA tool highly relevant.^23^

## Methods

A mixed-method study was conducted in a sequential explanatory design combining a quantitative and qualitative approach.^24^ Quantitative data was first collected through a cross-sectional survey, supplemented with qualitative data from cognitive interviews evaluating the survey questions and answers. We first analyzed the response patterns to the questionnaire items and then evaluated respondents’ understanding and interpretation of the questions to improve validity. The findings of the quantitative study were used to draft the interview guide. When integrating the findings, we compared the results of both approaches and analyze points of convergence, divergence, and complementarity. Additionally, the results of the qualitative approach were used to further interpret and explain quantitative findings.

### Quantitative approach

Our VHA tool was integrated into a cross-sectional survey among parents of newborn children using a one-off questionnaire.^25^ Parents were recruited to participate in the study when their child was approximately 2 weeks old. During a routinely scheduled home visit, a child and youth healthcare (CYH) professional gave an information leaflet about the study. Parents were eligible to participate in the period before their child could receive the first vaccination of their immunization schedule, typically starting at the age of 3 months. Hereby, we aimed to reach parents close to their decision-making stage. Prior to inclusion, informed consent was obtained. Participation was incentivized by raffling off gift vouchers. Parents were recruited in collaboration with six regional CYH centers across the Netherlands from December 2021 to December 2022. These six centers covered a wide geographical area of both urban and rural parts of the Netherlands. Each of these centers recruited parents through their multiple affiliate offices located in different communities with varying socioeconomic status. In this manner, we aimed to recruit a diverse study population.

We based the VHA tool on the most common and pragmatic approach described in the literature, by asking parents to indicate their level of hesitancy in a direct and self-reported manner.^9^ In our search for the appropriate wording, we adhered closely to the definition of VH, a state of indecisiveness regarding a vaccine decision, by including the element of ‘decision-making.’ The question to measure the degree of VH was formulated as followed ‘How do you feel about deciding on vaccinations for your newborn child?.’ Since, as in other languages^21^, there may not be a clear and unambiguous translation of ‘vaccine hesitancy’ in Dutch, we used five different wordings that resemble elements of hesitancy based on our prior qualitative analysis of the VH concept^9^: uncertainty (‘onzekerheid’), doubt (‘twijfel’), concerned (‘ongerustheid’), reluctance (‘aarzeling’), and indecision (‘besluiteloosheid’). Forward and backward translation was reviewed and discussed with a sworn translator. Participants were asked to answer the same VH question five times, each time with a differently worded answering option. They were instructed to score their answers on a 10-point Likert scale, where higher scores corresponded with a positive feeling (very certain, not doubtful, not concerned, not reluctant, and nor indecisive). For participants who were unable to answer, an opt-out option was provided. The question and the five answering options were pilot tested by five parents and professionals and found to be clear and understandable. The testing results were not included in the final results. The original and translated question and answering options are enclosed in supplementary file A.

Additionally, we included the validated short Parental Attitudes to Childhood Vaccines (PACV-5) scale, to compare its score with the results of our five differently worded answering options on hesitancy.^26^ We selected this scale as this is often used in the context of childhood vaccinations and also to validate a self-reported hesitancy method.^27^ The PACV-5 was forward and backward translated by two independent sworn translators. A sum score of the five PACV items was calculated, ranging between 0 and 10 and categorized in low (0–4), medium (5–6), and high (7–10) levels of hesitancy.^28^

We conducted a statistical analysis using SPSS software, with responses from participants who completed 75% of the questionnaire. In a first step, we evaluated to what extent the five answering options were similarly used by respondents and whether one or more option(s) was/were superior. To start with, we compared the mean and standard deviations of the five differently worded answering options to assess whether similar or different answers were given. Next, we calculated inter-item correlation coefficients and mean differences between the answers given using the five different hesitancy wordings. Then, a Cronbach’s alpha was calculated to evaluate the internal consistency of the five answering options. Subsequently, we compared each different hesitancy wording score with the PACV-5 score by calculating correlation coefficients. The level of correlation was interpreted as an indicator of vaccine hesitancy.

Once (a) superior answering option(s) was/were found, we subsequently determined appropriate cutoff values for hesitancy for the selected answering option in our VHA tool. For this purpose, we specifically used one of the five items of the PACV-5, the item on self-reported hesitancy: ‘Overall how hesitant about childhood shots would you consider yourself to be?.’ Categorical answering options used for this item are as follows: Not at all hesitant/not too hesitant/not sure/somewhat hesitant/very hesitant.’ Through visual inspection, we compared how the Likert scale scores of the VHA tool corresponded with the different PACV item answering categories. We additionally performed a receiver operating characteristic (ROC) curve analysis to identify a cutoff value with optimal sensitivity and specificity levels. For this analysis, we used the VHA score and a (medium or high) hesitancy defined by a PACV-5 score of 5 or higher.^26^

### Qualitative approach

We aimed to validate our quantitative findings on the different hesitancy wordings through a qualitative study. As will be described in the results section, the majority of the parents participating in the quantitative part of this study were highly educated. To evaluate whether the proposed VHA tool is also appropriate for parents with lower education levels, cognitive qualitative interviews were conducted in this target group.^29^ This validation study was embedded in a qualitative study on decision-making about childhood vaccination among lower educated parents. We purposively sampled parents of young children (<18 months old) with a lower education level to evaluate the VHA tool in this specific group. Parents were recruited through four CYH centers in both rural and urban areas across the Netherlands. Eligible parents were approached by a member of the research team in-person. A brief explanation of the study was provided, and a leaflet was handed over with information about the study, suitable for people with lower health literacy skills. When parents indicated willingness to participate, they were contacted by phone within one week to answer questions about the study. Interviews were scheduled for the period from July to December 2023. Prior to the start of the interview, written informed consent was obtained.

We aimed to include 10–12 parents and if necessary more until we reached data saturation. These interviews were conducted in a different time frame and through different CYH centers as compared to the quantitative study to avoid recruiting a parent who was already familiar with our VHA tool.

During an interview with one member of the research team (TW), participants were shown the question and answering options (as used in our quantitative approach) and encouraged to read them aloud and to explain what they thought was meant and why. They were also asked how they would score each of the five differently worded answering options, how they understood the different options, and which worded option they preferred and why. Finally, participants were asked to suggest wording alterations to improve the clarity of the question and answering options. The interview guide is enclosed in supplementary file B.

The interviews were transcribed verbatim and analyzed using ATLAS.ti. Additionally, one researcher (DB) listened to the recordings to hear if participants dropped long pauses or had difficulty reading aloud, indicating difficulty with comprehension. Audible comprehension issues were converted into codes. The coding framework consisted of positive and negative comments, interpretations, difficulties, preferences, and audible elements. Subsequently, these were categorized into four main themes related to 1) the question, 2) the five different answering options, 3) Likert scale, and 4) totality of question and answers. The findings were discussed by three members of the research team (MH, JH, DB).

### Ethical considerations

Participation in the study was considered to constitute a minimal burden for participants and not to be associated with risks. The Medical and Ethical Review Board of the Radboud University Medical Center concluded that both stages of the study were exempted from full review (registration numbers: 2021–13071 and 2023–16280).

## Results

### Quantitative approach

We received 600 responses to our questionnaire, of which 533 were at least 75% completed. As one participant did not complete the five differently worded VH answering options, 532 responses remained in our analysis. The mean age of the participants was 33 y (standard deviation (SD) 4.2 y), the majority (80%) was female, and 47% of the respondents were parents to a firstborn child. As for the level of education, 85% of the respondents were highly educated, and 15% had a lower education level.

### Comparison of the five differently worded VH answering options

We found that 314 (59%) of the participants gave exactly the same score to each of the five different hesitancy answering options. We then compared how participants responded to the five differently worded VH answering options on the 10-point Likert scales and found that the mean scores per type of phrasing ranged from 8.86 to 9.10 and SDs ranged from 1.37 to 1.48 (Table 1). These small variations indicate that the scores given to each VH response phrasing were very similar. Additionally, we calculated per respondent the difference in scores between each of the five options and the mean difference ranged from 0.01 to 0.2 (not shown in Table 1), again pointing at very similar responses. Subsequently, we found that the five options were all positively correlated with each other, with Pearson correlation coefficients ranging from 0.686 to 0.909 (*p* < .001) (Table 1). The Cronbach’s alpha for the five scale scores was 0.95, which again indicates that respondents answered the five answering options in a very similar manner. Taken together, the questions were answered for each of the five options in a very similar manner, and based on the analysis so far, we could not identify a superior option.

**Table 1.** Mean score and standard deviation of likert scores for each individual VH response phrasing, inter-item correlation between VH response phrasings and correlation with the PACV-5 score.

VH response phrasing	Mean	SD	Cronbach’s alpha	Inter-item correlation					Correlation PACV-5 score
				Uncertainty	Doubt	Concerned	Reluctance	Indecision	
Uncertainty	9.03	1.371	0.95	/	–	–	–	–	−0.489*
Doubt	9.05	1.390		0.853*	/	–	–	–	−0.525*
Concerned	8.86	1.419		0.735*	0.844*	/	–	–	−0.439*
Reluctance	9.06	1.392		0.782*	0.909*	0.854*	/	–	−0.515*
Indecision	9.10	1.484		0.686*	0.770*	0.712*	0.786*	/	−0.442*

VH = vaccine hesitancy, SD = standard deviation, PACV-5 = parental attitudes about childhood vaccination 5-item scale. A *p*-value < .001 is indicated with an asterisk (*).

Finally, we compared the scores given to the differently worded VH answering options with the PACV-5 sum score. The PACV-5 sum scores ranged from 1 to 10, indicating, respectively, low to high hesitancy levels, whereas a higher score on our VH items indicated lower hesitancy levels. We found all five scores to be negatively and significantly correlated with the PACV-5 sum score, Pearson correlation coefficients ranging from −0.439 to −0.525 (*p* < .001) (Table 1).

For our further analysis, we selected the vaccine hesitancy translation with the highest level of correlation with the PACV-5 score, resembling ‘doubt’ (correlation coefficient −0.525, *p* < .001). From this point onwards, this will be referred to as the VHA tool.

### Cutoff values

In a second step, we determined cutoff values for our VHA tool to distinguish between parents with and without VH. The majority (72,9%) scored 9 or 10, corresponding with ‘no hesitancy.’ A proportion of 27,1% of participants scored 8 or lower indicating some level of vaccine hesitancy. The distribution of score is shown in supplementary file C. We compared these scores with the answer to the PACV-5 question ‘Overall how hesitant about childhood shots would you consider yourself to be? (not at all hesitant/not too hesitant/not sure/somewhat hesitant/very hesitant’) (Table 2). The vast majority (88%) of participants who indicated ‘not at all hesitant’ on this question also scored 9 or 10 in our vaccine hesitancy instrument. Also, the majority (83%) of participants who indicated ‘somewhat’ or ‘very hesitant’ scored between 1 and 8 on our vaccine hesitancy scale. Participants indicating ‘not too hesitant’ scored either 9 or 10 (38%) or between 1 and 8 (62%).

**Table 2.** Comparison between the answers to PACV-5 question ‘overall how hesitant about childhood shots would you consider yourself to be?’ and corresponding likert score of the VHA tool.

			Vaccine Hesitancy Assessment (VHA) tool			
			Some degree of VH (score 1 t/m 8)		No VH (score 9 and 10)	Total
PACV-5 Question: Overall how hesitant about childhood shots would you consider yourself to be?	Not at all hesitant	No		46	335	381
		%		12%	88%	100%
	Not too hesitant	No		77	48	125
		%		62%	38%	100%
	Don’t know	No		1	1	2
		%		50%	50%	100%
	Somewhat hesitant	No		17	3	20
		%		85%	15%	100%
	Very hesitant	No		3	1	4
		%		75%	25%	100%
Total		No		144	388	532
		%		27%	72%	100%

PACV-5 = parental attitudes about childhood vaccination 5-item scale; VH = vaccine hesitancy.

The ROC curve analysis was performed to evaluate binary classification of VH based on the VHA score. We found an area under the curve (AUC) of 0.83 (95% CI 0.76–0.90) indicative of good discriminative ability. At a threshold of 8.5, we found a good balance of a sensitivity of 77% and a specificity of 74%. These findings supported the visual inspection of the comparison shown in Table 2. Based on these results, we identified a cutoff value of 8.5, where scores 1 to 8 correspond with some degree of hesitancy and scores 9 and 10 with no VH. The distribution of scores ranging from 1 to 8 was skewed and therefore did not allow for a further subdivision into groups with different hesitancy levels.

### Qualitative approach

We included 12 parents with a practical education level, 10 females and 2 males. Their ages ranged from 23 to 41 y (mean 31 y) and the age of their youngest child ranged from 2 to 18 months old (mean 5 months old). We established data saturation, as the last interviews did not provide any new insights.

When participants were asked to evaluate the question ‘How do you feel about deciding on vaccinations for your newborn child?,’ most participants indicated that this question was easy to understand. When read aloud, no hiccups or delays were audible. However, some participants noted that they had to read the question a few times before they understood what was meant and that the wording used might be difficult for others. Participants indicated that, based on the phrase ‘How do you feel … ?,’ they were expecting an open-ended answering option rather than a Likert-scale. They suggested including the answering option already in the question, for example ‘do you doubt?’ In this way, they thought it would be more clear to others.

Next, participants reviewed the five differently worded answering options with the 10-point Likert scale, with lower scores corresponding with a negative answer and higher scores with a positive answer. In general, participants indicated that the options were quite similar and to their understanding, the same was meant. They did, however, prefer some options over others:

*Very uncertain – not uncertain*: Generally, this option was perceived as very clear and matching the question. One participant mentioned a negative association of uncertainty with failure. Four participants indicated a clear preference for this option.

*Very doubtful – not doubtful*: This was perceived as very clear as well and described as ‘the best fit’ and five participants indicated a clear preference for this option. One participant mentioned that this wording gave her an unpleasant feeling and was not sure if this wording would be appropriate

*Very reluctant – not reluctant*: Most participants felt quite strongly that this was not an appropriate use of language. They mentioned that this was not used in contemporary language anymore, it is difficult to understand and ‘looked strange.’ Some participant left a long pause and none of the participants preferred this option.

*Very indecisive – not indecisive*: Also here, participants indicated that this was too difficult to understand, not often used and less suitable. One participant feared that people would get confused and would not be able to tell the difference between very and not indecisive. None of the participants preferred this option.

*Very concerned – not concerned*: Participants indicated that this option did not match the question. This wording was associated with ‘fear of vaccination,’ and one participant worried that this would evoke hesitancy. Two participants dropped long pauses reading aloud. None of the participants preferred this option.

In summary, the first two options (uncertainty and doubt) were viewed as most suitable, clear, and easy to understand words to indicate hesitancy. The other three options (reluctant, indecisive, concerned) were perceived as difficult, uncontemporary use of language, unclear and not recognized as words describing hesitancy. The slight majority preferred the option ‘doubt,’ although many indicated that this was quite similar to ‘uncertainty.’ Illustrative quotes are presented in Table 3.

**Table 3.** Illustrative quotes.

	Quote
Question wording	‘Yes, this is the kind of question that makes me think: huh? How do you feel about deciding? … I have to read this question a few times before I think: oh yeah, what do they mean by this? I might phrase it differently. But then I can’t say how.’ [ID7] ‘I would indeed ask it more colloquially.’ [ID5]
Uncertain	’[This evokes] … that you might fail, that you think you have failed.’[ID1] ‘Yes indeed I think, how do you feel about vaccinating, then very uncertain, I think that describes it well. … So in terms of word choice I think that is the best fit.’ [ID3]
Doubt	‘I think that very certain and very doubtful/not doubtful are clearer for most people and for most target groups. I think the more difficult the words you use or the more polite … , it seems as if the words [reluctant, indecisive, concerned] are becoming more polite or more formal, and that it becomes difficult for many people to really understand the question.’ [ID2] ‘That because of that … Very doubtful/not doubtful is part of making a decision. And being certain or uncertain is a feeling. And to me, that is not part of making a decision, so to speak. … This [doubt] fits better in my opinion’ [ID12]
Reluctance	‘Reluctance is a word that I think is hardly used in the Netherlands anymore. At least not in the younger vernacular. Reluctance. No, that is just doubt.’ [ID5] ‘Yes, I would definitely, [reluctance] that is a really difficult word, for sure. … Or I look at my husband, for example, who came to the Netherlands when he was 8 years old, so he lacks some basics of the Dutch language. Then this is just not easy.’ [ID6]
Indecision	‘This one is simply out of the question for me, indecisive. … That can really—There are really people who will just not understand that. Who can’t link that to the question … Indecisive. I can’t remember ever using the word indecisive in my twenty-five years of experience.’ [ID9]
Concerned	‘I think that during pregnancy you are really uncertain at times and certainly about what is happening to your body: you change so much, your hormones. Then I think that ‘very concerned’ or ‘not concerned,’ well, then I think: yes. Maybe I would think: is there any reason to be worried about vaccination? That you start having doubts, so to speak.’ [ID10] ‘Concerned. This is [about] someone who is afraid of the risk of vaccinations.’ [ID5]
Likert score	‘Then I definitely give myself a 9, as in that I feel very confident about that. But I can never say 100%.’ [ID3] ‘Although I always find it difficult with these kind of questions to answer on a scale from 1 to 10. Then you don’t really have … you do give an answer but I don’t really think that you can make it very clear what you actually think about it. In that case I would give a 10, because I am certain that I will do it [vaccination] and have done it.’ [ID2]

Additionally, participant gave their views on how they would interpret and use the Likert scale. A number of participants indicated that they would never score the maximum (10) because they could never be a 100% sure. For them, the score 9 resembled their highest level of certainty, and in this case, lowest level of vaccine hesitancy. We also came across dissenting views, as participants mentioned that it is difficult to give a numerical score to a subject that they would rather answer as an open question and suggested to leave space for a comment or explanation. For example, one participant argued that indicating hesitancy on a scale was not a good method, as it did not represent her views in a good way.

### Integration of quantitative and qualitative results

The quantitative and qualitative parts of this study together describe the development and the evaluation of content, construct, and face validity of the VHA tool. Our quantitative results indicated that the differently worded VH alternatives are answered very similarly and as one construct, demonstrated by a high Cronbach's alpha of 0.95. One wording alternative (‘doubt’) performed slightly better. We integrated these findings in the interviews by evaluating how parents perceived these words and whether their understanding of these words was indeed similar. The interviews provided complementary insights into conceptualizations and preferences of lower educated parents. Although they confirmed that the wording alternatives had a similar meaning, they also indicated their preferences of which wording alternative captured hesitancy most clearly. When comparing the preferred alternative with the quantitative findings, the results converged. The integrated results point out that the wording alternative in Dutch resembling ‘doubt’ is the preferred use of language to assess VH, based on comparison with a gold standard, the PACV-5, and cognitive interviews with parents.

Furthermore, we compared the quantitative findings of the visual inspection of the VHA score and corresponding answer to the PACV-5 hesitancy question and ROC curve analysis, which suggested a cutoff at 8.5. The qualitative study provided explanatory information on which meaning parents give to a certain Likert score. They clearly indicated that a score 9 or 10 corresponded with not experiencing hesitancy. Some argued that they would never indicate a 10, since they could never be 100% sure. These complementary findings endorse the cutoff for VH (≤8) that was proposed based on the quantitative assessment. When comparing the results of the quantitative and the qualitative analysis further, the findings diverged and gave no basis to further categorize scores into different degrees of VH.

The views and preferences of the parents participating in this study guided further modification and optimization of the VHA tool. We suggest minor adaptation and simplification of the wording of the question itself. This includes adopting experiencing hesitancy in the question instead of ‘How do you feel’ to give more direction. We furthermore suggest using a formulation that hints toward rating on a scale, for example by the use of ‘to what extent.’ When using the VHA in the future, we would suggest that the wording of the question be changed to ‘To what extent do you *hesitate* when deciding on your child’s vaccination?.’ The suggested wording for future use of the VHA, both English and Dutch is enclosed in supplementary file D.

## Discussion

We demonstrate the development and validation of the VHA tool in the non-English language through a people-centered approach. Parent’s input guided our evaluation of the most appropriate reflection of ‘vaccine hesitancy’ in Dutch language building on our prior qualitative analysis of the VH concept.^13^ When applying the VHA tool, parents who report VH during decision-making more often do not vaccinate their children according to the recommended schedule later on.^25^ This highlights the importance of approaching parents early-on during decision-making to assess and address their hesitancy.

The strength of this study lies in combining different methodological approaches. The cognitive interviews proved to be a valuable method to generate complementary user-based insights on psychometric properties that shaped the development of our VHA tool. Furthermore, the integration of quantitative data with qualitative insights, particularly from a lower-educated panel, not only enriched our understanding of the findings but also ensured usability of the VHA tool in a broad target group with different literacy levels. Overall, this contributed to the validity and reliability of the VHA tool. This study also has several limitations. We approached parents of newborn infants prior to their scheduled routine vaccinations and assumed, for practical reasons, that they would be close to their decision-making stage. As we did not validate this, it is possible that some parents had made their decision much earlier and this may have led to a recall bias. In the future research, we would recommend validating the stage of decision-making. Furthermore, repeated measures with the VHA tool would give further insight into how VH evolves over time.

Another limitation is that in the quantitative part of this study, parents with a lower level of education were underrepresented, and therefore this sample may not fully represent the spectrum of vaccine hesitancy in the general population. As a mitigation strategy and to improve the overall representability, we only included parents with a lower level of education in the qualitative part of this study. Additionally, the results of the qualitative part of this study may have been influenced by a desirability bias. However, we prioritized the needs of lower educated parents, to develop a tool suitable for a broad group of parents with different health literacy skills. When designing future studies on vaccine hesitancy, including further evaluation of the VHA tool, enabling participation of a diverse population should be a priority.

According to widely used translation tools, including the Cambridge Dictionary, Google, and DeepL Translate, the primary translation for hesitancy in Dutch is the word ‘aarzeling,’ which can also be translated as ‘reluctance.’ We included this wording alternative in our study and participants described that it ‘looked strange,’ ‘is an old-fashioned word, that is not used anymore nowadays’ and ‘is difficult to understand.’ This example illustrates that merely translating hesitancy instruments to a different language may not lead to an equivalent meaning and similar level of usability.^30^ When translating VH terminology, careful consideration of the context in which it is used, is warranted. Therefore, when applying the VHA in another language and context, additional cross-cultural validation is needed to ensure applicability in different contexts and meaningful comparisons across different contexts.^31^ Our mixed methods approach can serve as an example in this regard and could be applied in other cultural contexts to cross-culturally validate the VHA tool.

In our sample, we utilized 10-point Likert scales to rate VH, and we were able to identify that scores ‘9’ and ‘10’ corresponded with not experiencing hesitancy in decision-making. Based on these findings, we pragmatically proposed a cutoff between people without hesitancy (score >8) or with some level of hesitancy (score ≤8). This was supported by the results of the ROC curve analysis. Nevertheless, whether this threshold performs at a similar level in different populations should be further evaluated. Importantly, during the cognitive interviews, participants described that they felt it was difficult to rate their answers on a Likert scale. Prior research showed that for people with lower literacy, a response scale with fewer categories, preferably three, would be more suitable.^32^ To further improve our VHA tool we recommend, on the one hand, to differentiate between higher and lower levels of VH and, on the other hand, reconsider the number of response categories to further facilitate using the tool for those with lower literacy skills. To improve the inclusivity of the VHA tool, it would be important to pilot the use of 3- or 5-point Likert scales in future research projects.

In a broader context of declining vaccination coverage, waning confidence and spillover of beliefs about COVID-19 vaccination, it is important to take a pro-active approach. Uptake of vaccination is an important indicator, but can only be measured once a decision about vaccination is made. Shifting focus to stages preceding vaccination uptake would add value and could contribute to a more holistic view on hesitancy and vaccination. The VHA tool could be used for this purpose, as it merely aims to identify VH, a state of indecision regarding a vaccination decision. Currently, interventions developed to address hesitancy such as educational tools^33^ or decision support tools^34^ are mostly evaluated by their impact on vaccine uptake.^35^ It would, however, be very informative to also evaluate how such interventions impact hesitancy itself.

Based on our findings, we conclude that our VHA tool is a robust and practical method to assess VH in a self-reported manner with one item. We propose to use the VHA tool to monitor VH at population level and among (future) parents when deciding about childhood vaccinations. This timing is important, because when shifting our focus to parents who have not decided and have not formed their views yet, a window of opportunity may arise for decisional support interventions.

## Supplementary Material

Supplemental file A.docx

Supplemental file C.docx

Supplemental file B.docx

Supplementary file D_clean.docx
