# Supplementary material for: Developing a practical tool for measuring parental vaccine hesitancy: A people-centered validation approach in Dutch
Source: Hum Vaccin Immunother. 2025 Feb 17;21(1):2466303. doi: 10.1080/21645515.2025.2466303 (PMC11834527; doi:10.1080/21645515.2025.2466303)
Supplement: Supplemental file A.docx [file KHVI_A_2466303_SM0110.docx]

**Supplement A: original and translated question and answering options**

Original question and answering options in Dutch:

1. Hoe voelt u zich in het beslissen over de vaccinaties voor uw pasgeboren kind?

Heel onzeker 1 - 2 - 3 - 4 - 5 - 6 - 7 - 8 - 9 - 10 Heel zeker

1. Hoe voelt u zich in het beslissen over de vaccinaties voor uw pasgeboren kind?

Veel twijfel 1 - 2 - 3 - 4 - 5 - 6 - 7 - 8 - 9 - 10 Geen twijfel

1. Hoe voelt u zich in het beslissen over de vaccinaties voor uw pasgeboren kind?

Heel ongerust 1 - 2 - 3 - 4 - 5 - 6 - 7 - 8 - 9 - 10 Heel gerust

1. Hoe voelt u zich in het beslissen over de vaccinaties voor uw pasgeboren kind?

Veel aarzeling 1 - 2 - 3 - 4 - 5 - 6 - 7 - 8 - 9 - 10 Geen aarzeling

1. Hoe voelt u zich in het beslissen over de vaccinaties voor uw pasgeboren kind?

Heel besluiteloos 1 - 2 - 3 - 4 - 5 - 6 - 7 - 8 - 9 - 10 Niet besluiteloos

Original question and answering options translated to English:

1. How do you feel about deciding on vaccinations for your newborn child?

Very uncertain 1 - 2 - 3 - 4 - 5 - 6 - 7 - 8 - 9 - 10 Very certain

1. How do you feel about deciding on vaccinations for your newborn child?

Very doubtful 1 - 2 - 3 - 4 - 5 - 6 - 7 - 8 - 9 - 10 Not doubtful

1. How do you feel about deciding on vaccinations for your newborn child?

Very concerned 1 - 2 - 3 - 4 - 5 - 6 - 7 - 8 - 9 - 10 Not concerned

1. How do you feel about deciding on vaccinations for your newborn child?

Very reluctant 1 - 2 - 3 - 4 - 5 - 6 - 7 - 8 - 9 - 10 Not reluctant

1. How do you feel about deciding on vaccinations for your newborn child?

Very indecisive 1 - 2 - 3 - 4 - 5 - 6 - 7 - 8 - 9 - 10 Not indecisive
