# Supplementary material for: Developing a practical tool for measuring parental vaccine hesitancy: A people-centered validation approach in Dutch
Source: Hum Vaccin Immunother. 2025 Feb 17;21(1):2466303. doi: 10.1080/21645515.2025.2466303 (PMC11834527; doi:10.1080/21645515.2025.2466303)
Supplement: Supplemental file C.docx [file KHVI_A_2466303_SM0109.docx]

Supplement C: distribution of VHA tool scores

| VHA score | Frequency (%) |
| --- | --- |
| 1 | 2 (0.4%) |
| 2 | 1 (0.2%) |
| 3 | 3 (0.6%) |
| 4 | 3 (0.6%) |
| 5 | 8 (1.5%) |
| 6 | 5 (0.9%) |
| 7 | 30 (5.6%) |
| 8 | 92 (17.2%) |
| 9 | 106 (19.8%) |
| 10 | 282 (52.7%) |
| Total: | 532 (100%) |
