# Supplementary material for: Developing a practical tool for measuring parental vaccine hesitancy: A people-centered validation approach in Dutch
Source: Hum Vaccin Immunother. 2025 Feb 17;21(1):2466303. doi: 10.1080/21645515.2025.2466303 (PMC11834527; doi:10.1080/21645515.2025.2466303)
Supplement: Supplemental file B.docx [file KHVI_A_2466303_SM0108.docx]

**Supplement B: interview guide**

Introduction

Thank you for participating in this study. In this part of the interview, we would like you to have a look at a question for parents who are deciding about vaccination. We are curious to hear what you think and how we could do better. When you are ready, I will show you the question and answers. The question remains te same, but the answering option changes.

Question

- Would you read the question aloud?
- How do you feel about this question?
- What does this question evoke?
- What do you think is meant?

Five different answering options (presented one-by-one)

- What do you think about this answering option and why?
- What does this evoke?
- How would you score this answer on the scale and why?
- Which answer option best describes what you think and why?
- Which answer best fits the question and why?
- Which option do you prefer and why?

Suggestions

- Would you ask the question differently and how?
- How would you have done it differently?
- What would work better for you or someone else?
