# Supplementary material for: Developing a practical tool for measuring parental vaccine hesitancy: A people-centered validation approach in Dutch
Source: Hum Vaccin Immunother. 2025 Feb 17;21(1):2466303. doi: 10.1080/21645515.2025.2466303 (PMC11834527; doi:10.1080/21645515.2025.2466303)
Supplement: Supplementary file D_clean.docx [file KHVI_A_2466303_SM0107.docx]

Supplement D: suggested wording for future use of the vaccine hesitancy assessment (VHA) tool

Suggested wording in Dutch:

In hoeverre twijfelt u bij de keuze over de vaccinaties voor uw kind?

Veel twijfel 1 - 2 - 3 - 4 - 5 - 6 - 7 - 8 - 9 - 10 Geen twijfel

Suggested wording translated to English:

To what extent do you hesitate when deciding on your child's vaccination?

Very hesitant 1 - 2 - 3 - 4 - 5 - 6 - 7 - 8 - 9 - 10 Not hesitant
